# COVID-19 in cancer patients: patient characteristics and outcomes in the post-COVID-19 vaccination period

**DOI:** 10.55730/1300-0144.5744

**Published:** 2023-10-12

**Authors:** Gülşen İSKENDER, Duygu MERT, Göknur YAPAR TOROS, Funda YILMAZ, Ersin BOZAN, Semra TUNÇBİLEK, Ömür Berna ÇAKMAK ÖKSÜZOĞLU, Fevzi ALTUNTAŞ, Mustafa ERTEK

**Affiliations:** 1 Department, of Infectious Diseases and Clinical Microbiology, Ankara Oncology Training and Research Hospital, University of Health Sciences, Ankara, Turkiye; 2 Department of Infectious Diseases and Clinical microbiology, Ankara Etlik City Hospital, Ankara, Turkiye; 3 Division of Medical Oncology, Erzurum City Hospital, Erzurum, Turkiye; 4 Department of Hematology and Bone Marrow Transplantation Center, Ankara Oncology Training and Research Hospital, Ankara, Turkiye; 5 Department of Medical Oncology, University of Health Sciences, Ankara, Turkiye; 6 Department of Hematology and Bone Marrow Transplantation Center, Ankara Oncology Training and Research Hospital, University of Health Sciences, Ankara, Turkiye

**Keywords:** Cancer patients, COVID-19, COVID-19 vaccination

## Abstract

**Background/aim:**

It wasaimed herein to investigate coronavirus disease (COVID-19) in cancer patients and compare hematological and solid organ cancer patients in terms of the course and outcome of this disease.

**Materials and methods:**

Data from cancer patients with laboratory-confirmed COVID-19 infection were analyzed retrospectively. Risk factors for poor prognosis and the effect of vaccination on the clinical outcomes of the patients were evaluated.

**Results:**

A total of 403 cancer patients who were diagnosed with COVID-19 between March 1st, 2021, and November 30th, 2022, were included, of whom 329 (81.6%) had solid and 74 (18.4%) had hematological cancers. Hospitalization and intensive care unit (ICU) admission rates were significantly higher in the hematological cancer patients compared to the solid organ cancer patients (73.0% vs. 35.9%, p< 0.001 and 25.7% vs. 14.0%, p= 0.013, respectively). The COVID-19**-**related case fatality rate (CFR) was defined as 15.4%, and it was higher in the hematologicalcancer patientsthan inthe solid organ cancer patients (23.0% vs. 13.7%, p= 0.045) and was higher in patients with metastatic/advanced disease compared to the other cancer stages (p< 0.001). In the solid organ cancergroup, hospitalization, ICU admission, and the COVID-19 CFR were higher in patients with respiratory and genitourinary cancers (p< 0.001). A total of 288 (71.8%) patients had receivedCOVID-19 vaccination; 164 (56.94%) had≤2 doses and 124 (43.06%) had≥3 doses. The hospitalization rate was higher in patients with ≤2 doses of vaccine compared to those with ≥3 doses (48.2% vs. 29.8%,p= 0.002). Patients with COVID-19**-**related death had higher levels of leucocyte, neutrophil, D-dimer, troponin, C-reactive protein (CRP), procalcitonin, and ferritin and lower levels of lymphocyte than the survivors. In the logistic regression analysis,the risk of COVID-19**-**related mortality was higher in the hematological cancer patients(OR:1.726), those who were male (OR:1.757), and with the Pre-Delta/Delta variants (OR:1.817).

**Conclusion:**

This study revealed that there is an increased risk of COVID-19-related serious events (hospitalization, ICU admission, or death) in patients with hematological cancerscompared with those who have solid organ cancers. It wasalso shown that receiving ≥3 doses of COVID-19 vaccine is more protective against severe illness and the need for hospitalization than ≤2 doses.

## 1. Introduction

The new severe acute respiratory syndrome coronavirus type 2 (SARS-CoV-2) andassociated infectious coronavirus disease (COVID-19) have affected the world and caused a large number of deaths since it was first identified in late 2019. Globally, as of August 9th, 2023, there have been more than 769 million confirmed cases of COVID-19, including more than 6.9 million deaths^1^. On May 5th, 2023, the end of COVID-19 as a public health emergency was declared by the head of the UN World Health Organization (WHO).However, this does not mean that the disease is no longer a global threat and concerns also remain about the potential for more potent variants of the virus to emerge 2,3. Global efforts to combat SARS-CoV-2 have led to the rapid developmentof multiple vaccines and the trial of a variety of therapeutic agents. At this stage, global vaccination remains an important intervention in controlling infection and minimizing its spread in the community^4^.

Studies have shown that cancer patients are very susceptible to SARS-CoV-2 infection and the risk of serious illness and death is high in these patients [1]. In addition, cancer patients often have a prolonged period of COVID-19, causing the disruption of their cancer treatment and jeopardizing its success [2]. Moreover, it was reported that the immune response to the vaccines is lower in cancer patients. especially in thosewith hematological malignancies and hematopoietic cell transplantation recipients (HCT) [3,4]. This has led to the recommendation of bivalent/polyvalent vaccines and a higher number of primary and booster doses, especially in cancer patients compared to the normal population [5,6]^5^. In Türkiye, vaccination against SARS-CoV-2 began on January 14th, 2021, with the CoronaVac vaccine (inactivatedvero cell vaccine) as a decision bythe Turkish Ministry of Health and the BNT162b2 (Pfizer-BioNTech, mRNA) vaccine has been available since April 2021^6^. As of the date of this article, bivalent COVID-19 vaccines are not yet available in Türkiye.

To date, most of the studies evaluating the course of COVID-19 in cancer patients in Türkiyewere conducted during the prevaccination period. In the currentstudy,it wasaimed to investigate the baseline characteristics of hematologic and solid organ cancer patients with COVID-19, the risk factors for severe disease and poor prognosis, and the effect of SARS-CoV-2 vaccination and vaccine doses on the clinical outcomesin an oncology training and research hospital.

## 2. Materials and methods

### 2.1. Study population

Hematological and solid organ cancer patients with laboratory-confirmed SARS-CoV-2 infection at an oncology training and research hospital, between March 1st, 2021 and November 30th, 2022,were evaluated retrospectively. All of the patients had COVID-19-positive reverse transcription polymerase chain reaction (RT-PCR) nasopharyngeal and oropharyngeal smear samples.

Inclusion criteria were: 1) being≥18 years of agewithfollow-up in our hospital fora diagnosis of hematological or solid organ malignancy in the 5years preceding the COVID-19 diagnosis(including those actively receiving anticancer treatment and those in clinical follow-up),2)havingSARS-COV-2 PCR-positive resultson the sample taken from the oro/nasopharynx, and 3) having clinical and laboratory data that could be accessed for at least 3 months after the COVID-19diagnosis.

Exclusion criteria were: 1) having no malignancy,2) having malignancy but being <18 years of age,3) having malignancy and beingSARS-COV-2 PCR-negativeor the tests had not beenperformed, but there wereclinical and laboratory findings compatible with COVID-19, and 4) having malignancy butclinical and laboratory findings could not be accessed for up to 3 months after the COVID-19diagnosis.

The data of the patients were obtained from the electronic medical records of our hospital. The patients’ age, sex, comorbidities, malignancy stage at COVID-19 diagnosis (newly diagnosed untreated, active under treatment, metastatic/advanced, relapsed/refractory, and remission/cured), type of last anticancer treatment (conventional chemotherapy, proteasome inhibitors, targeted therapy, and hormonal therapy), date of last anticancer treatment, COVID-19 vaccination status, laboratory findings related to COVID-19 at the time of diagnosis, hospital and intensive care unit (ICU) admission, and outcomes (COVID-19-related mortality) were recorded.

The COVID-19**-**related case fatality rate (CFR) wasdefined as the rate of death among patients with cancer and COVID-19. Attributable or contributable deaths were defined on the basis of the subjective judgment of the local physician.

Classification of the date of diagnosis according to the periods in which the SARS-CoV-2 variants appeared; pre-Delta/Delta: from the start of the pandemic to the end of November 2021, Omicron and its subvariants: from December 2021 to the end of the study.

The study objectives were to1) determine the effect of the demographic characteristics, comorbidities, cancer type, cancer stage, and anticancer treatments on COVID-19 severity and outcomes, 2) determine the relationship between the first laboratory findings and COVID-19 severity and outcomesand make a comparison of the hematological and solid organ cancers in this respect,3) compare the clinical characteristics of patients with and without COVID-19-related mortality, and 4) evaluate the effect of vaccination against SARS-CoV-2 and the number of vaccine doses on disease progression and outcomes in cancer patients.

### 2.2. Statistical analysis

Statistical analysis was done using IBM SPSS Statistics for Windows 27.0 (IBM Corp., Armonk, NY, USA). Frequency tables and descriptive statistics were used to interpret the findings. Nonparametric methods were used for measurement values that did not fit the normal distribution. In accordance with nonparametric methods, the Mann–Whitney U test (Z-table value) was used to compare the measured values of 2independent groups. Pearson-χ2 crosstabs were used to evaluate the relationship between 2qualitative variables. Binary logistic regression (LR) analysis (backward LR method) was used to define the factors affecting the risk of COVID-19-related mortality. p< 0.05 was considered statistically significant. As a result of the power analysis using the G*Power 3.0.1 program; with a medium effect size (d=0.5), 5% margin of error, and 80% power, a total of at least 128 samples was found to be sufficient (with at least 64 samples in each group) for the study.

The study design was approved by the Ministry of Health of the Turkish Republic (2022-04-25 T14-07-34) and the Ankara Oncology Training and Research Hospital Clinical Research Ethics Committee (Date: 12/18/2022, Decision No: 2022-12-180).

## 3. Results

A total of 403 PCR-positive COVID-19 patients with cancer were evaluated during the study period. Of these patients, 329 (81.6%) had solid organcancer and 74 (18.4%) had hematological cancer. The demographic and characteristics of the patients are shown in Table 1. The overall mean age was 59.89± 13.59 years;60.61± 12.75 years in the solid organ cancer group and 56.72± 16.58 years in the hematological cancer group. There was no significant difference between the groups in terms of the mean age (p= 0.219). Female sex(57.1%) was significantly predominant in the solid organ cancer group, while male sex (58.1%) was in the hematological cancer group (p< 0.001) (Table 1).

In terms of the SARS-CoV-2 variant periods; 150 patients (37.2%) were diagnosed with COVID-19 in the pre-Delta/Delta period and 253 (62.8%) patients were diagnosed in the Omicron period.

Considering the number of COVID-19 episodes (first infection and reinfections),in the hematological and solidorgan cancer groups, 6 (8.1%) and 33 (10.0%) patients experienced ≥2 COVID-19 reinfections, respectively.

The cancer type distribution in the solid organ cancer patients (329) was as follows:breast (96/329 patients, 29%), gastrointestinal (81/329 patients, 24.6%), genitourinary (58/329 patients, 17.6%), respiratory (41/329 patients, 12.5%), and others (53/329 patients, 16%) (cancer types with a small number of patients). The cancer type distribution in the hematological cancer patients (74) was as follows:leukemia (28/74 patients, 37.83%), non-Hodgkin’s lymphoma (22/74 patients, 29.72%), multiple myeloma (17/74 patients, 23.00%), and Hodgkin’s lymphoma (7/74 patients, 9.45%).

The most common comorbidities were diabetes mellitus and hypertension in both groups (Table 1).

In terms of the malignancy stage,the solid organ cancer patients were predominantly in the metastatic/advanced stage (41%) and those with hematological cancer were predominantly in the active under treatment stage (32.4%) (Table 1).

Hospitalization and ICU admission rates were significantly higher in the hematological cancer patients compared to the solid organ cancer patients(73.0% vs. 35.9%, p< 0.001 and 25.7% vs. 14.0%, p = 0.013, respectively) (Table 1).

Statistically significant differences were found between the groups in terms of the white blood cell (WBC), lymphocyte, neutrophil, and platelet (PLT) counts performed at the time of COVID-19 diagnosis; these values were lower in thehematological cancer patientscompared to the solid organ cancer patients(p< 0.05) (Table 2). There was no significant difference between the groups in terms of the D-dimer, troponin, CRP, procalcitonin, and ferritin values (p> 0.05) (Table 2).

There was no significant relationship between the cancer types and hospitalization/ICU admission in the hematological cancer group(p> 0.05). However, in the solid organ cancer group,the hospitalization rate was higher for those withgenitourinary (29/58 patients, 50.0%) and respiratory system (17/41 patients, 41.5%) cancers (p< 0.001), and the ICU admission rates were also higher in those with respiratory (9/41 patients,22.0%) and genitourinary (11/58 patients,19%) cancers (p< 0.001).

There were no significant differences between the groups in terms of comorbidities and the number of COVID-19 episodes and time (months) between the last chemotherapy and the development of COVID-19 (p> 0.05).

In terms of COVID-19-related mortality, the overall COVID-19-related case fatality rate (CFR) in the cancer patients was 15.4% (62/403 patients). The overall COVID-19 CFR was higher in the hematologicalcancer group compared to the solid organ cancer group(23.0% vs. 13.7%, p= 0.045) (Table 1). Overall, COVID-19-related mortality was significantly higher in the cancer patients with metastatic/advanced disease (33/62 patients, 53.2%) (Table 3). A comparison of the patients with and without COVID-19-related mortality in terms of the laboratory findings at the time of COVID-19 diagnosis showed that the WBC count, neutrophil count/percentage, D-dimer, troponin, CRP, procalcitonin, and ferritin levels were significantly higher and the lymphocyte count/percentage wassignificantly lower in patients with COVID-19-related mortality (Table 4). As a result of the backward LR analysis, it was determined that the cancergroup, male sex, and COVID-19 variantperiod were important predictors affecting COVID-19-related mortality. The risk of COVID-19-related mortality was 72.6% higher in patients with hematologicalcancer compared to those with solid organ cancer(p= 0.039, OR:1.726), 75.7% higher in males compared to females (p= 0.045, OR:1.757), and 81.7% higher in the Pre-Delta/Delta period compared to the Omicron period (p= 0.033, OR:1.817). Considering the relationship of age with hospitalization, ICU admission, and COVID-19-related mortality,in the hematologicalcancer patients, these rates were higher in the ≥60 age group and in the solid organ cancer patients, they were higherin the ≥70 age group, but these differences were not statistically significant (p> 0.05). No statistically significant correlation was found between COVID-19-related mortality and comorbidities or the number of COVID-19 episodes (p> 0.05) (Table 3). No statistically significant correlation was found between COVID-19-related mortality and the cancer type in the hematological cancer group(p> 0.05). In the solid organ cancer group, COVID-19-related mortality was significantly higher in those with respiratory and genitourinary systemcancers compared to those with breast and gastrointestinal cancers (17.1% and20.7% vs. 6.2% and 5.2%) (p< 0.001).

In terms of the COVID-19 vaccination status,288 (71.8%) of the cancer patients were vaccinated; 164 (56.94%) had ≤2 doses and 124 (43.06%) had ≥3 doses. The number of vaccine doses and their distribution in thecancergroups are given in Table 1.The overall hospitalization rate was significantly higher in those who received ≤2 doses (79/164 patients: 48.2%) compared to ≥3 doses (37/124 patients,29.8%, p= 0.002).Overall, patients who received ≤2 doses of vaccine compared to ≥3 doses had higher rates of ICU admission (29/164 patients, 17.7% vs. 13/124 patients,10.5%) and COVID-19 CFR (26/164 patients, 15.9% vs. 14/124 patients, 11.3%), but these differences were not statistically significant (p> 0.05).

Inthesolid organ cancergroup, the hospitalization rate was significantly higher in those who had ≤2 doses of COVID-19 vaccine (54/133 patients, 40.6%) than in those who had ≥3 doses (24/106 patients,22.6%) (p= 0.003). In this cancer group, patients who received ≤2 doses compared to ≥3 doses had higher rates of ICU admission (13.5% vs. 9.4%) and COVID-19 CFR (12.0% vs. 10.4%), but these differences were not statistically significant.

In the hematological cancer group, patients who received ≤2 doses compared to ≥3 doses had higher rates of hospitalization (80.6% vs. 72.2%), ICU admission (35.5% vs. 16.7%), and COVID-19 CFR (32.3% vs. 16.7%), but these differences were not statistically significant.

There were no significant differences between the groups in terms of being vaccinated against COVID-19, vaccine doses, or time from the last vaccine dose to the diagnosis of COVID-19 (Table 1).

A comparison of the Pre-Delta/Delta and Omicron variant periods is given in Table 5.In the pre-Delta/Delta period, most of the patients had ≤2 doses of vaccination (71/150 patients, 86.6%), and in the Omicron period, most of the patients had ≥3 doses (113/253 patients, 54.9%), and these differences were statistically significant (p< 0.001). The hospitalization and ICU admission rates were higher in the pre-Delta/Delta period compared to the Omicron period (54.7% vs. 35.6%, p< 0.001 and 22.0% vs. 16.6%, p= 0.014 respectively). The COVID-19 CFR also was higher in the pre-Delta/Delta period compared to the Omicron period (20.7% vs. 12.3%, p= 0.024).

When the relationship betweenthe vaccine doses andhospitalization and mortality was examined in the Omicron period, it was found that the rate of hospitalization, ICU admission, and COVID-19 CFR were higher in patients who had≤2 doses of COVID-19 vaccine compared to those who had≥3 doses, but these differences were not statistically significant (p> 0.05) (Table 6).

Overall, no statistically significant relationship was found between the types of anticancer treatments (conventional chemotherapy, proteasome inhibitors, targeted therapy, and hormonal therapy) and the rates of hospitalization, ICU admission, and COVID-19-related mortality (p> 0.05).

## 3. Discussion

Evaluated herein were 403 PCR-positive COVID-19 cancer patients. The patients were mainly in the metastatic or active cancer stages and solid organ cancers were predominant (81.6%). Moderate to severe COVID-19with the needed for hospitalization/ICU admission and COVID-19-related CFR were higher in the hematologicalcancer group compared to the solid organ cancer group. Overall, the hospitalization rate was significantly higher in those who had≤2 doses of COVID-19 vaccine. In addition, patients who had≤2 doses of COVID-19 vaccine had higher rates of ICU admission and mortality from COVID-19.

COVID-19 disproportionately affects immunocompromised cancer patients when compared to the general population. A delay in the diagnosis and treatment of malignancies during the pandemic process also played a role in the worsening of COVID-19 outcomes in these patients [7,8]. Due to serious illness concerns in these patients, adherence to isolation precautions and vaccination schedules is strongly recommended by experts [9].

Studies have indicated that malignancy in comparison to other comorbidities is associated with a higher risk of admission to the ICU, invasive ventilation, and death from COVID-19 [10].It has been reported that mortality rates secondary to COVID-19 in cancer patients are approximately 30% (reported as around 40% in the first wave of COVID-19 and around 25% in subsequent waves),whichis at least 5times higher than inpatients without malignancy [1,7,11]. When studies evaluating COVID-19 in patients with solid organ cancers were examined,in a multicenter study from India,the COVID-19-related CFR was determined as 14.4% in solid cancer patients(likelihood ratio of 4.4,p= 0.030), while in a study from Belgium,the 30-day in-hospital COVID-19-related mortality rate was reported as 31.7% in patients with solid cancer and 20.0% in patients without cancer [12,13]. In patients with hematological cancer, a study from Türkiye stated that the rates of severe/critical illness and CFR were significantly higher in hematological cancer patients in comparison withnoncancerous patients (18.9% vs. 11.5% and 13.8% vs. 6.5%, respectively) [14]. In another study conducted in the pre-COVID-19 vaccination period, thetotal CFR in patients with and without active hematologic malignancies was 22.2% and 4.2%, respectively [15].

Studies have also shown that severe diseasewith the needed forhospitalization/ICU admission and COVID-19-related mortality rates were higher in hematological cancer patients in comparison to solid organ cancer patients [12,16,17]. The overall COVID-19-related CFR in the cancer patients in the current study was 15.4% and the rates of hospitalization, ICU admission, and CFR were higher in the hematological cancer patientscompared to thesolid organ cancer patients.

Current data have indicated that lymphoproliferative disorders, in particular non-Hodgkin’s lymphoma, chronic lymphocytic leukemia, and multiple myeloma, are particularly associated with a higher risk of SARS-CoV-2 infection. In studies from Türkiye,it was reported that non-Hodgkin’s lymphoma, myelodysplastic syndrome, and myeloproliferative neoplasms were the most common hematological malignancies in patients with COVID-19 [14,18]. Certain tumor types have been found to be associated with particularly poor outcomes with COVID-19. Considering the COVID-19-related mortality,acute leukemia, high-risk myelodysplastic syndrome, chimeric-antigen-receptor-T therapies and HCT were found to be associated with high risk [11,19]. In terms of solid organ cancers,in a systematic review and metaanalysis that comprised theresults of COVID-19 in cancer patients from 110 studies across 10 countries,genitourinary, gastrointestinal, and respiratory system cancers accounted for 91.6% of all reported solid cancers in these patients [1]. While the highest COVID-19-related mortality rate in solid cancers was found in respiratory system cancers, potentially due to the reduced respiratory reserve, different rates have been reported for other cancer types. Some studies have found a moderately high mortality rate associated with COVID-19 in genitourinary, female genital tract, and breast cancers, but there are also studies showing that these types of cancer carry a low risk of death [20,21]. In the presentstudy population,the most common cancer types in the hematological cancer group were leukemia (37.83%) and non-Hodgkin’s lymphoma (29.72%), while in the solid organ cancer group, these were breast (29.2%) and gastrointestinal (24.6%) cancers. A significantly high rate of ICU admission and COVID-19-related mortalitywasfound in the patients with respiratory (22.0% and 17.1%) and genitourinary cancers (19.0% and 20.7%) (p< 0.001) compared to the other types of solid organ cancers. There was no significant relation between hospitalization, ICU admission, and COVID-19-related mortality and thecancer types in the hematological cancer group (p> 0.05).

Considering the comorbidities that may affect the COVID-19 course and outcome, studies have shown that older age, male sex, cardiovascular and metabolic comorbidities, specially diabetes mellitus, and active or uncontrolled malignancy represent the main risk factors in both hematological and solid organ cancer patients [12,19]. In the European Hematology Association Survey (EPICOVIDEHA),conducted by Pagano et al.[11], it was found thatage, active malignancy, chronic cardiac, liver, and renal diseases, smoking history, and ICU stay were correlated with mortality in COVID-19 patients with hematological malignancy. Although diabetes mellitus and hypertension were the most common comorbidities in both the hematological and solid organ cancer patients in the currentstudy, no significant relationship was found between the comorbidities and COVID-19-related mortality rates. It wasfound that the metastatic/advanced malignancy stage and male sex werecorrelatedwith mortality. The age cut-off for worse prognosis in COVID-19 was found to be 65 years in some studies, while others found it to be 75 years [20,21]. Although not statistically significant, it wasfound herein that older age (≥60 years in the hematological cancer patients and ≥70 years in the solid organ cancerpatients) wasassociated with higher rates of hospitalization/ICU admission and COVID-19-related mortality.

Studies on the outcomes of COVID-19 in cancer patients have shown that, as in the normal population, the presence of leukocytosis, lymphocytopenia, thrombocytopenia and neutropenia were associated with poor prognosis and increased mortality rate [11].Since these laboratory findings can also develop in non-COVID-19 cancer patients,especially hematological and HCT patients, the coexistence of cancer and COVID-19 mayplay a role in worsening laboratory findings. COVID-19 has been found to be associated with significant venous and thrombotic events (VTEs) with the relationship termed COVID-19-associated coagulopathy. Patients with cancer are also particularly at risk of thrombotic complications. An elevated blood D-dimer level has been shown to predict a higher risk of VTE. D-dimer has the potential to also serve as a risk stratification tool in COVID-19 [7,12].Aworse prognosis was detected in the hematological cancer patients compared to those with solid organ cancer, as well as lower WBC, lymphocyte, neutrophil, and platelet counts at the time of COVID-19 diagnosis in these patients. In the analysis of the relationship between these laboratory findings and poor prognosis,it wasfound thatpatients with COVID-19-related mortality had significantly higher rates oflymphocytopenia, leukocytosis, and neutrophilia, and higher levels of D-dimer, troponin, CRP, procalcitonin, and ferritin levels than the survivors.

Immunosuppressive cytotoxic chemotherapies are thought to increase the risk of severe COVID-19 in cancer patients. This risk varies according to the different tumor types and therapeutic approaches [1,6,22]. However, the impact of immunotherapy (IO) or additional treatments that affect the immune system has not been fully established. In a registry-based retrospective cohort study in patients with cancer and COVID-19, it was found that the administration of systemic anticancer therapies, especially IO (due to immune system activation), in cancer patients with baseline immunosuppression was associated with severe clinical outcomes and the development of cytokine storm [6]. No significant correlation was detected between the types of anticancer treatments and COVID-19 course and outcome in the current study. This may have been due to aninsufficient number of patients included in the study for this analysis and/or the heterogeneity of the patient population.

Since the beginning of the pandemic, many countries have carried out studies to develop a vaccine against SARS-CoV-2. Vaccination has shown high efficacy in reducing community transmission, hospitalization, and deaths due to severe COVID-19 in the general population. Cancer patients, due to the high risk of developing SARS-CoV-2 infection and severe disease, have priority in vaccination [19]. The WHO recommends that countries vaccinate at least 70% of their population, 100% of healthcare workers, and the most vulnerable groups, including people over 60 and those who are immunocompromised or have underlying diseases^7^. It should be noted that cancer patients may have a lower immune response to vaccines, attributed to the discordant antibody and cellular responses due to the cancer itself or immunosuppressive therapies [3,4,19,23,24].In addition, it was determined that the SARS-CoV-2 antibody response decreased 3to 5months after the second vaccine dose and a stronger serological response emerged after the third dose of the vaccine in cancer patients. It has beenemphasized that more SARS-CoV-2 vaccine doses are strongly associated with a decrease in the severity of infection in this patient population [5,25–27]. Previously, the Centers for Disease Control and Prevention (CDC) recommended a 3-dose primary mRNA vaccine series and accelerated booster dose in immunocompromised patients^8^. In the CDC’s last update (July 17th, 2023),it was recommended that people who are moderately or severely immunocompromised receive 2doses of the updated COVID-19 vaccine, 2or more months apart, regardless of whether they have received any of the original COVID-19 vaccines^4^. On June 30th, 2021, the Ministry of Health of the Republic of Türkiyeannounced that it had beendecided to give the third dose vaccine to the >50 age group, patients with underlying comorbidities, including immunosuppressives and healthcare professionals^6^.In Türkiye, approximately 85.70% of the population has had2doses of COVID-19vaccine and around 45% have had3 doses (as of August 13th, 2023)^9^. In the currentstudy population, 71.8% of the COVID-19 cancer patients were vaccinated; 56.94% had ≤2 doses and 43.06% had≥3 doses. The overall hospitalization rate was significantly higher in those who had≤2 doses of vaccine. In addition, although not statistically significant, the rates of ICU admission and COVID-19-related CFR were higher in those who had ≤2 doses of vaccine compared to those who had ≥3 doses. Studies have indicated that the Omicron variant was associated with less severe disease than the Delta variant, but still resulted in substantial morbidity and mortality [28]. Studies have shown that levels of neutralizing antibodies and protective effectiveness against severe COVID-19 are higher after 3 doses of the original COVID-19 vaccine compared to 2 doses, and that these effects are generally lower against the Omicron variant compared to the previous variants [29]. In the presentstudy,duringthe pre-Delta/Delta period, most of the patients had≤2 doses of vaccineand duringthe Omicron period, most had ≥3 doses. Moderate to severe diseasewith the needed forhospitalization/ICU admission and COVID-19-related mortality rates were higher during the pre-Delta/Delta period compared to the Omicron period. In this case, we are faced with the following questions: 1) Were these findings due to the less severe disease caused by the Omicron variant? Or 2) Were they due to the higher number of vaccine doses during this period? To understand this, the relationship betweenvaccine doses andhospitalization and mortality during the Omicron period was evaluated and it was found that hospitalization, ICU admission, and COVID-19-related mortality were higher in patients who had≤2 doses in this period as well(although this was not statistically significant). Therefore, it can be said that the severity of COVID-19 is lower in patients who have had ≥3 dosesof vaccine than in patients who have had ≤2 doses for each variant period.

This study revealed that there is an increased risk of COVID-19-related serious events (hospitalization, ICU admission, or death) in patients with hematological cancer compared with solid organ cancer. It was also shown that, forcancer patients,having≥3 doses of COVID-19 vaccine is more protective against severe disease and the need for hospitalization than ≤2 doses.

The limitations of thisstudy were that it was conducted in a single-center and it was done retrospectively.

The strength of thestudy,as wasmentioned in the introduction,is that to date, most of the studies evaluating the course of COVID-19 in cancer patients inTürkiyewere performed in the pre-COVID-19 vaccination period. However,herein, the course and prognosis of COVID-19 in the post-COVID-19 vaccination period was evaluated and compared in patients with hematological and solid organ cancers who were followed-up in a comprehensive oncology hospital. It was also attempted to reveal the effect of COVID-19 vaccination and the number of vaccine doses on the severity of the disease and mortality.

In conclusion, many studies conducted around the world. support the effectiveness of booster doses of vaccine against SARS-CoV-2 variants, including Omicron, especially in vulnerable individuals such as the elderly, immunocompromised, or those who have concomitant comorbidities. Revealing the clinical course of COVID-19 in cancer patients and the effect of vaccination on it with real-life data will guide health policy makers in creating appropriate vaccination programs and the preparation of plans to increase compliance with vaccination in these special patients.

## Figures and Tables

**Table 1:** Characteristics of the cancer patients with COVID-19.

Malignancy group	Hematological (n= 74)		Solid organ (n= 329)		Total (n= 403)		Statistical analysis^1^
Characteristics	n	(%)	n	(%)	n	(%)	
**Sex**							
Female	31	(41.9)	188	(57.1)	219	(54.3)	**p= 0.017**
Male	43	(58.1)	141	(42.9)	184	(45.7)	
**Malignancy stage**							
Active under treatment	24	(32.4)	82	(24.7)	106	(26.1)	p= 0.185
Metastatic/advanced	5	(6.8)	136	(41.0)	141	(34.7)	**p< 0.001**
Remission/cured	21	(28.4)	30	(9.0)	51	(12.6)	**p< 0.001**
Relapsed/refractory	17	(23)	15	(4.5)	32	(7.9)	**p< 0.001**
Newly diagnosed untreated	7	(9.4)	69	(20.8)	76	(18.7)	**p= 0.022**
**Comorbidities** 2							
DM	19	(37.3)	81	(30.7)	100	(31.7)	p= 0.849
CRD	1	(2.0)	6	(2.3)	7	(2.2)	p= 0.779
HT	18	(35.3)	108	(40.8)	126	(40.0)	p= 0.154
CF	-	-	1	(0.4)	1	(0.3)	p= 0.635
CVD	7	(13.6)	29	(11.0)	36	(11.4)	p= 0.861
CPD	3	(5.9)	24	(9.1)	27	(8.6)	p= 0.314
Others	3	(5.9)	15	(5.7)	18	(5.7)	p= 0.849
**COVID-19 episodes**							
First infection	68	(91.9)	296	(90.0)	364	(90.3)	p= 0.613
≥2 re-infections	6	(8.1)	33	(10.0)	39	(9.7)	
**COVID-19 vaccination**							
Unvaccinated	24	(32.9)	89	(27.1)	113	(28.2)	p= 0.324
Vaccinated	49	(67.1)	239	(72.9)	288	(71.8)	
**COVID-19 vaccine dose**s							
1	4	(8.2)	22	(9.2)	26	(9.0)	p= 0.374
2	27	(55.1)	111	(47.4)	138	(47.9)	
3	10	(20.4)	77	(32.2)	87	(30.3)	
≥4	8	(16.3)	29	(12.2)	37	(12.8)	
**Time from last vaccine to COVID-19**							
≤6 months	37	(75.5)	157	(65.7)	194	(67.4)	p= 0.182
>6 months	12	(24.5)	82	(34.3)	94	(32.6)	
**Hospitalization**							
No	20	(27.0)	211	(64.1)	231	(57.3)	**p< 0.001**
Yes	54	(73.0)	118	(35.9)	172	(42.7)	
**ICU admission**							
No	55	(74.3)	283	(86.0)	338	(83.9)	**p= 0.013**
Yes	19	(25.7)	46	(14.0)	65	(16.1)	
**COVID-19 CFR** 3							
No	57	(77.0)	284	(86.3)	341	(84.6)	**p= 0.045**
Yes	17	(23.0)	45	(13.7)	62	(15.4)	

1 p-value denotes the Pearson-χ2 test,

2 DM: diabetes mellitus, CRD: chronic renal disease, HT: hypertension, CF: cardiac failure, CVD: cardiovascular disease, CPD: chronic pulmonary disease,

3 CFR: case fatality rate.

**Table 2:** Laboratory findings at the time of COVID-19 diagnosis.

Malignancy group variables	Hematological (n= 74) X̄ ± SD^1^ Median[IQR]^2^	Solid organ (n= 329) X̄ ± SD Median[IQR]	Total (n= 403) X̄ ± SD Median[IQR]	Statistical analysis 3

WBC^4^	9.10±34.4	7.58±5.6	7.87±15.9	**p< 0.001**
	4.7[4.5]	6.5[4.4]	6.2[4.3]	

Lymphocyte	1.65±5.5	1.43±2.6	1.47±3.4	**p< 0.001**
	0.7[1.1]	1.1[1.1]	1.1[1.1]	

Lymphocyte(%)	27.45±26.1	22.02±15.2	23.07±17.9	p= 0.599
	21.5[28.7]	20.1[17.9]	20.2[19.6]	

Neutrophil	3.59±3.1	5.85±5.9	5.41±5.5	**p< 0.001**
	3.2[4.5]	4.4[4.1]	4.2[3.8]	

Neutrophil (%)	76.24±97.4	69.41±33.6	70.74±52.3	p= 0.869
	70.9[38.3]	686[19.8]	68.9[21.8]	

PLT^5^	153.08±98.5	233.74±91.4	218.08±96.6	**p< 0.001**
	121.0[169.3]	226.0[151.0]	212.0[160.0]	

D-dimer	4288.75±13269.44	4449.56±7771.63	4387.80±10186.45	p= 0.066
	1150.0[1455.0]	1400.0[3415.0]	1310.0[1895.0]	

Troponin	24.79±34.1	49.10±157.3	42.95±131.1	p= 0.604
	8.2[21.8]	8.9[17.8]	8.3[18.5]	

CRP^6^	77.98±70.6	127.46±67.8	113.56±76.2	p= 0.639
	59.7[120.2]	43.5[104.9]	48.8[109.7]	

PCT^7^	1.55±5.6	3.12±11.8	2.58±10.1	p= 0.773
	0.19[0.6]	0.19[0.7]	0.19[0.6]	

Ferritin	723.56±629.69	928.67±1601.02	868.01±1384.36	p= 0.525
	578.9[1114.1]	423.5[706.3]	446.0[732.0]	

1 S.D: standard deviation

2 IQR: inter quantile range,

3 p-value denotes Mann–Whitney U test,

4 WBC: white blood cell count,

5 PLT: platelet count,

6 CRP: C-reactive protein,

7 PCT: procalcitonin.

**Table 3:** Relationship between COVID-19-related mortality and the characteristics of the cancer patients.

COVID-19-related mortality	No (n= 341)		Yes (n= 62)		Total (n= 403)		Statistical analysis^1^
Characteristics	n	(%)	n	(%)	n	(%)	
**Malignancy stage**							
Active under treatment	95	(27.6)	11	(17.7)	106	(26.1)	p= 0.096
Metastatic/advanced	108	(31.4)	33	(53.2)	141	(34.7)	**p= 0.001**
Remission/cured	45	(13.1)	6	(9.7)	51	(12.6)	p= 0.443
Relapsed/refractory	23	(6.7)	9	(14.5)	32	(7.9)	**p= 0.037**
Newly diagnosed untreated	73	(21.2)	3	(4.9)	76	(18.7)	**p= 0.002**
**Comorbidities** 2							
DM	79	(30.6)	21	(36.7)	100	(31.7)	p = 0.073
CRD	6	(2.3)	1	(1.8)	7	(2.3)	p= 0.935
HT	103	(39.9)	23	(40.4)	126	(40.0)	p= 0.282
CF	1	(0.4)	-	-	1	(0.3)	p= 0.669
CVD	29	(11.3)	7	(12.3)	36	(11.4)	p= 0.479
CPD	24	(9.3)	3	(5.3)	27	(8.6)	p= 0.524
Others	16	(6.2)	2	(3.5)	18	(5.7)	p= 0.607
**COVID-19 episodes**							
** First infection**	309	(90.6)	55	(88.7)	364	(90.3)	p= 0.641
** ≥2 re-infections**	32	(9.4)	7	(11.3)	39	(9.7)	

1 p value denotes the Pearson-χ2 and Mann-Whitney U tests,

2 DM: diabetes mellitus, CRD: chronic renal disease, HT: hypertension, CF: Cardiac failure, CVD: cardiovasculer disease, CPD: chronic pulmonary disease.

**Table 4:** Relationship between COVID-19-related mortality and the laboratory findings.

COVID-19-related mortality	No (n= 341) X̄ ± SD^1^ Median[IQR]^2^	Yes (n= 62) X̄ ± SD Median[IQR]	Statistical analysis^3^

Variables			

WBC^4^	6.64±4.8	14.11±37.3	**p< 0.001**
	6.0[4.0]	7.9[7.9]	

Lymphocyte	1.53±3.6	1.17±1.7	**p= 0.003**
	1.1[1.1]	0.8[0.9]	

Lymphocyte (%)	24.07±17.4	17.99±20.0	**p< 0.001**
	21.3[18.8]	10.2[20.5]	

Neutrophil	4.99±5.3	7.52±6.4	**p< 0.001**
	4.0[3.3]	6.3[6.5]	

Neutrophil (%)	68.45±48.7	82.34±67.0	**p< 0.001**
	67.7[20.3]	82.6[20.6]	

PLT^5^	219.56±117.5	210.57±166.6	p= 0.199
	216.0[153.0]	190.0[254.0]	

D-dimer	3984.45±10859.7	5385.00±8346.4	**p= 0.001**
	1150.0[1430.0]	2210.0[3970.0]	

Troponin	21.61±49.4	89.72±229.9	**p= 0.006**
	6.1[11.1]	17.3[42.9]	

CRP^6^	61.71±665.8	288.19±1189.8	**p< 0.001**
	31.5[91.0]	116.0[130.7]	

Procalcitonin	1.53±6.4	4.82±15.2	**p< 0.001**
	0.1[0.4]	0.4[3.2]	

Ferritin	581.55±660.4	2146.01±2632.6	**p< 0.001**
	402.5[546.3]	1132.0[1563.0]	

1 S.D: standard deviation,

2 IQR:Inter Quantile Range,

3 p value denotes Pearson-χ2 and Mann-Whitney U” tests,

4 WBC: white blood cell count,

5 PLT: platelet count,

6 CRP: C-reactive protein.

**Table 5:** Vaccine doses, hospitalization, ICU admission, and mortality during the COVID-19 periods.

COVID-19 periods	Pre-Delta/Delta (n= 150)		Omicron (n= 253)		Total (n= 403)		Statistical analysis^1^
Variables	n	(%)	n	(%)	n	(%)	
COVID-19 vaccine doses							
≤2	71	(86.6)	93	(45.1)	164	(56.9)	**p< 0.001**
≥3	11	(13.4)	113	(54.9)	124	(43.1)	
Hospitalization							
No	68	(45.3)	163	(64.4)	231	(57.3)	**p< 0.001**
Yes	82	(54.7)	90	(35.6)	172	(42.7)	
ICU admission							
No	117	(78.0)	221	(87.4)	338	(83.9)	**p< 0.014**
Yes	33	(22.0)	32	(12.6)	65	(16.1)	
COVID-19 CFR^2^							
No	119	(79.3)	222	(87.7)	341	(84.6)	**p= 0.024**
Yes	31	(20.7)	31	(12.3)	62	(15.4)	

1 p-value denotes the Pearson-χ2 test.

2 CFR: case fatality rate.

**Table 6:** Association of the vaccine doses with hospitalization and mortality during the Omicron perio**d**.

Vaccine doses	≤2 doses (n= 93)		≥3 doses (n= 113)		Statistical analysis^1^
Variables	n	(%)	n	(%)	
Hospitalization					
No	57	(61.3)	81	(71.7)	p= 0.115
Yes	36	(38.7)	32	(28.3)	
ICU admission					
No	80	(86.0)	103	(91.2)	p= 0.245
Yes	13	(14.0)	10	(8.8)	
Overall COVID-19-related CFR^2^					
No	80	(86.0)	102	(90.3)	p= 0.345
Yes	13	(14.0)	11	(9.7)	

1 p-value denotes the Pearson-χ2 test,

2 CFR: case fatality rate.

## Footnotes

1 World Health Organization (2023). WHO Coronavirus Disease (COVID-19) Dashboard [online]. Website https://covid19.who.int [accessed 9 August 2023].

2 UN News Global perspective Human stories (2023). WHO chiefdeclaresendto COVID-19 as a global healthemergency[online]. Website https://news.un.org/en/story/2023/05/1136367 [accessed 20 May 2023].

3 World Health Organization (2023). Coronavirus disease (COVID-19) situation reports [online]. Website https://www.who.int/emergencies/diseases/novel-coronavirus-2019/situationreports [accessed 3 August 2023].

4 Center for Disease Control and Prevention (2023). Stay up to date with COVID-19 vaccines [online]. Website https://www.cdc.gov/coronavirus/2019-ncov/vaccines/stay-up-to-date.html [accessed 17 July 2023].

5 CentersforDisease Control andPrevention (2023). InterimClinicalConsiderationsforUse of COVID-19 VaccinesCurrentlyAuthorized in the United States[online]. Website https://www.cdc.gov/vaccines/covid-19/clinical-considerations/covid-19-vaccines-us.html [accessed 31 May 2023].

6 T.C.Sağlık Bakanlığı COVID-19 Aşısı Bilgilendirme Platformu (2021). Aşı uygulanacak grup sıralaması [online]. Website https://covid19asi.saglik.gov.tr/TR-77707/asi-uygulanacak-grup-siralamasi.html [accessed 31 May 2021].

7 World Health Organization (2022). Coronavirus disease/ COVID-19 vaccines [online]. Website https://www.who.int/emergencies/diseases/novel-coronavirus-2019/covid-19-vaccines [accessed 22 July 2022].

8 CentersforDisease Control andPrevention (2021). COVID-19 VaccinesforModeratelytoSeverelyImmunocompromised People [online]. Websitehttps://www.cdc.gov/coronavirus/2019-ncov/vaccines/recommendations/immuno.html. [accessed 3 August 2023].

9 T.C.Sağlık Bakanlığı COVID-19 Aşısı Bilgilendirme Platformu (2023). Günlük aşı verileri [online].Website https://covid19asi.saglik.gov.tr/ [accessed 13 Agust 2023].
